# Detection of subclinical cardiotoxicity in sarcoma patients receiving continuous doxorubicin infusion or pre-treatment with dexrazoxane before bolus doxorubicin

**DOI:** 10.1186/s40959-019-0056-3

**Published:** 2020-01-02

**Authors:** Jieli Li, Hui-Ming Chang, Jose Banchs, Dejka M. Araujo, Saamir A. Hassan, Elizabeth A. Wagar, Edward T. H. Yeh, Qing H. Meng

**Affiliations:** 1 Department of Laboratory Medicine, The University of Texas MD Anderson Cancer Center, Houston, TX USA; 2 Center for Precision Medicine, Department of Medicine, University of Missouri School of Medicine, Columbia, MO USA; 3 Departments of Cardiology, The University of Texas MD Anderson Cancer Center, Houston, TX USA; 4 Department of Sarcoma Medical Oncology, Division of Cancer Medicine, The University of Texas MD Anderson Cancer Center, Houston, TX USA

**Keywords:** Cardiotoxicity, High sensitivity troponin T, Global longitudinal strain, Dexrazoxane, Continuous doxorubicin infusion

## Abstract

**Background:**

Continuous infusion of doxorubicin or dexrazoxane pre-treatment prior to bolus doxorubicin are proven strategies to protect against doxorubicin-induced cardiotoxicity. Recently, global longitudinal peak systolic strain (GLS) measured with speckle tracking echocardiography (STE) and high-sensitivity troponin T (hs-TnT) have been validated as sensitive indicators of doxorubicin-induced cardiotoxicity. Here, we asked whether changes in hs-TnT and/or GLS can be detected in patients who were treated with continuous infusion of doxorubicin or pre-treated with dexrazoxane followed by bolus doxorubicin.

**Methods:**

Twenty-nine patients with newly diagnosed sarcoma were assigned to receive either 72-h doxorubicin infusion or dexrazoxane pre-treatment before bolus doxorubicin. Eight patients received dexrazoxane pre-treatment; eleven patients received continuous doxorubicin infusion; ten patients crossed over from continuous infusion to dexrazoxane. Bloods were collected for hs-TnT at baseline, 24 h or 72 h after initiation of doxorubicin treatment in each chemotherapy cycle. All blood samples were assayed in batch using hs-TnT kit from Roche diagnostics. 2D Echo and STE were performed before doxorubicin, after cycle 3, and at the end of chemotherapy.

**Results:**

Seven patients in the cross-over group have at least one hs-TnT measurement between 5 ng/L to 10 ng/L during and after chemotherapy. Ten patients have at least one hs-TnT measurement above 10 ng/ml during and after chemotherapy (six in dexrazoxane group, three in continuous infusion group, one in cross-over group). The average hs-TnT level increases with each additional cycle of doxorubicin treatment. Eight patients had a more than 5% reduction in LVEF at the end of chemotherapy (four in dexrazoxane group, three in continuous infusion group, and one in cross-over group). Four out of these eight patients had a change of GLS by more than 15% (three in the dexrazoxane group).

**Conclusion:**

Elevation in hs-TnT levels were observed in more than 59% of patients who had received either continuous doxorubicin infusion or dexrazoxane pre-treatment before bolus doxorubicin. However, changes in LVEF and GLS were less frequently observed. Thus, continuous doxorubicin infusion or dexrazoxane pre-treatment do not completely ameliorate subclinical doxorubicin-induced cardiotoxicity as detected by more sensitive techniques.

## Introduction

In the era of targeted therapy, anthracyclines are still commonly used for the treatment of sarcomas, breast carcinomas, leukemia, and lymphomas [1]. Despite its efficacy in cancer therapy, the use of anthracyclines is limited by a dose-dependent cardiotoxicity, which can progress to severe cardiomyopathy without early recognition and treatment [1–6]. The most commonly used anthracycline is doxorubicin, which is usually given as a bolus infusion over 15 min [7]. Early retrospective studies showed that doxorubicin given in smaller doses caused less cardiotoxicity than the high-dose regimen; this is confirmed by using endomyocardial biopsy to detect cardiac damage [8]. It was postulated that oncologic efficacy correlated with the area under the plasma distribution curve, whereas peak plasma level is a better predictor of cardiotoxicity [7]. Continuous infusion of doxorubicin over 48 h or more have been shown to increase the cumulative doxorubicin dose that can be safely given to patients [7]. However, continuous infusion appeared not to be efficacious in preventing doxorubicin-induced cardiotoxicity in children with acute lymphocytic leukemia [9]. Doxorubicin intercalates DNA and covalently attaches to DNA double strand breaks via topoisomerase 2a, which is required for cell division [10]. In early studies, doxorubicin was shown to generate reactive oxygen species through redox regulation mediated by iron [11]. An iron chelator, dexrazoxane, was found to be efficacious in reducing doxorubicin-induced cardiotoxicity [12, 13]. However, recent literature suggests that dexrazoxane ameliorates doxorubicin-induced cardiotoxicity through a direct inhibition of topoisomerase 2b, not through its iron chelating activity [14, 15]. Dexrazoxane is the only FDA-approved therapy to protect against doxorubicin-induced cardiotoxicity in breast cancer patients. A large number of clinical trials have demonstrated the efficacy of dexrazoxane administration in ameliorating the development of doxorubicin-induced cardiotoxicity [13, 16–18]. Since sarcoma patients typically received higher dose of doxorubicin as compared to breast cancer patients, doxorubicin is usually given as a 72-h continuous infusion or pre-treated with dexrazoxane to reduce the incidence of cardiotoxicity at the University of Texas MD Anderson Cancer Center.

Cardiac troponins have served as an excellent biomarker of myocardial damage for more than three decades [19]. Recently, the high-sensitivity troponin assays have become available for detection of very low concentrations of circulating troponin [20]. A number of studies showed that cardiotoxicity could be predicted by elevation in high sensitivity troponin I (hs-TnI) in patients treated with trastuzumab [21], or anthracyclines [22]; Cardiotoxicity can also be predicted by high sensitivity troponin T (hs-TnT) level in breast cancer patients treated with anthracyclines and trastuzumab [23] or stem cell transplant patients treated with anthracyclines [24]. However, hs-TnT was not found to be a sensitive marker for late onset subclinical anthracycline-induced cardiotoxicity in long-term survivors of childhood cancer [25]. Global longitudinal strain (GLS) is regarded as more sensitive measure of left ventricular function [22]. GLS measurement increased diagnostic sensitivity and were associated with the risk of left ventricular dysfunction in patients treated with the anthracycline epirubicin [26]. However, high-sensitivity troponin or GLS has not been evaluated in patients who received cardio-protective measures, such as continuous doxorubicin infusion or dexrazoxane pre-treatment prior to bolus doxorubicin. In this study, we prospectively investigated whether hs-TnT assay and GLS can detect subclinical myocardial damage induced by doxorubicin in sarcoma patients who were treated with cardio-protective measures against doxorubicin-induced cardiotoxicity.

## Methods

Patients with newly diagnosed sarcoma undergoing doxorubicin treatment at the University of Texas MD Anderson Cancer Center were enrolled in this study. From June 2013 to February 2015, 29 patients, who fit the inclusion and exclusion criteria, were enrolled. Exclusion criteria included unstable coronary syndromes, decompensated heart failure, significant arrhythmias and severe valvular disease. We prospectively measured hs-TnT levels. All patients were assessed by electrocardiogram and echocardiography before treatment to exclude cardiac failure, cardiomyopathy, conduction disturbances, significant arrhythmias and severe valvar disease. Twenty-nine patients with newly diagnosed sarcoma were assigned to receive either 72-h doxorubicin infusion or dexrazoxane pre-treatment before bolus doxorubicin. Eight patients received dexrazoxane pre-treatment; eleven patients received continuous doxorubicin infusion; ten patients crossed over from continuous infusion to dexrazoxane due to development of systematic symptoms.

Bloods were drawn for hsTnT measurement before and at 24 or 72 h after doxorubicin treatment. Blood was collected into lithium heparin tube, centrifuged, and the plasma was removed and stored at − 80 °C. The hs-TnT concentrations were determined with high-sensitive cTnT reagents on Roche cobas e411 analyzer using electrochemiluminescence immunoassay (Roche Diagnostics, Indianapolis, IN, USA), with a lower detection limit of 5 ng/L and a reported 99th percentile value in apparently healthy individuals of 19 ng/L [27].

Doxorubicin-based chemotherapy cycles were given in 21 or 28 days cycles. Most patients received at least six cycles, some of them up to eight cycles. End of study is defined as the time when patient chemotherapy was completed. Elevated troponin is defined as hs-TnT > 5 ng/L. Echocardiography was performed after cycle 3 and at the end of chemotherapy. We measured both left ventricular ejection fraction (LVEF) and global longitudinal peak systolic strain (GLS) to assess left ventricular systolic dysfunction. Patients who received less than three cycles of doxorubicin were excluded from analysis.

The institutional review board of University of Texas MD Anderson Cancer Center approved the protocol, and all participating patients provided written informed consent.

### Statistical analysis

Continuous variables were expressed as mean ± standard deviation (SD) or mean ± standard error (SE). A *p* value < 0.05 was considered statically significant. All analyses were performed using one-way analysis of variance (ANOVA) by SPSS Statistics.

## Results

**Fig. 1:**
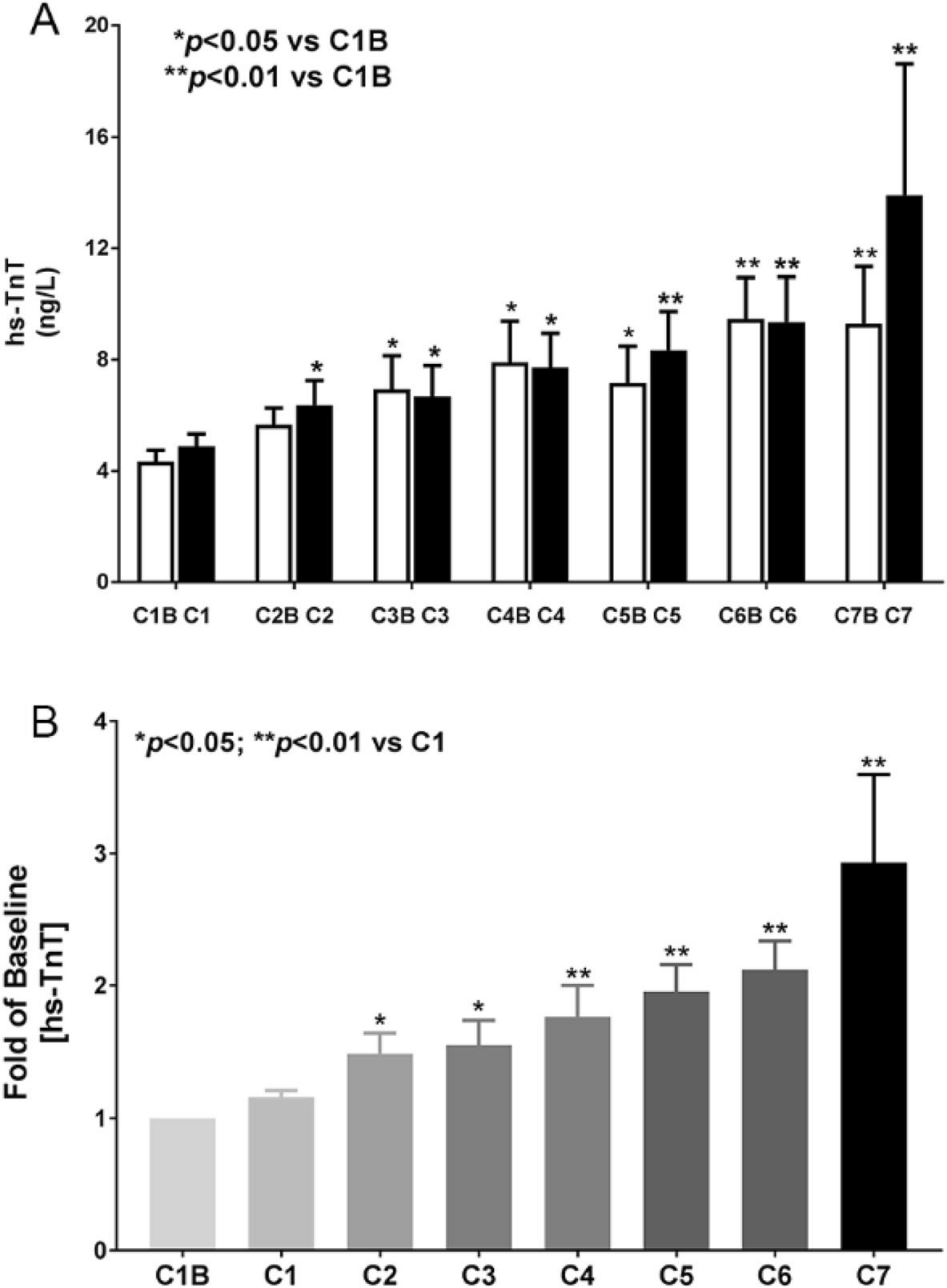
Time-line of hs-TnT results with doxorubicin continuous infusion or dexrazoxane pre-treatment. **a** Absolute value of hs-TnT with each chemotherapy cycle **b** Fold change of baseline of hs-TnT with each chemotherapy cycle. C1: cycle 1; C1B: cycle 1 baseline

Twenty-nine patients (16 females and 13 males), age 23 to 71 years old, were enrolled in this prospective study (Table 1). Ten patients crossed over from continuous infusion to the dexrazoxane group due to development of mucositis or other symptoms (Table 2). Seven patients in the cross-over group have at least one hs-TnT measurement between 5 ng/L to 10 ng/L during and after chemotherapy (Table 3). Ten patients have at least one hs-TnT measurement above 10 ng/ml during and after chemotherapy (six in dexrazoxane group, three in continuous infusion group, one in cross-over group). The average hs-TnT level increases with each additional cycle of doxorubicin treatment. The highest level of hs-TnT was observed in patients who have received 7 cycles of doxorubicin (Fig. 1).

**Table 1:** Baseline characteristics of patients

Protective strategy	Dexrazoxane	Continuous infusion	Crossover
Total Number	8	11	10
Age	25–69	24–71	23–66
Sex (M/F)	3/5	7/4	3/7

**Table 2:** Characteristics of Cross-over patients

Patient	Continuous infusion	Dexrazoxane	Reason for cross-over
1	Cycle 1	Cycle 2–4	Severe hemorrhoids
2	Cycle 1–3	Cycle 4	Mucositis
3	Cycle 1–6	Cycle 7–8	Concerns for cardiac toxicity
4	Cycle 1–3	Cycle 4–6	Mucositis
5	Cycle 1–2	Cycle 3–4	Mucositis
6	Cycle 1–6	Cycle 7–8	Concerns for cardiac toxicity
7	Cycle 1–2	Cycle 3–6	Mucositis
8	Cycle 1–3	Cycle 4–6	Mucositis
9	Cycle 1–3	Cycle 4–6	Rectal irritation
10	Cycle 1–2	Cycle 3–6	Septic Shock

Continuous infusion: doxorubicin 75 mg/m^2^ for 72 h

Dexrazoxane: dexrazoxane 750 mg/m^2^ followed by doxorubicin 75 mg/m^2^ over 15 min

**Table 3:** Patients with peak hs-TnT elevations

Protective strategy	Dexrazoxane	Continuous infusion	Crossover
Patient Number	8	11	10
Peak hS-TnT > 5 ng/ml	0	0	7
Peak hs-TnT > 10 ng/ml	6	3	1

Eight patients had a more than 5% reduction in LVEF at the end of chemotherapy (Table 4). Reduction of EF is not associated with any particular strategy for cardio-protection. Four out of these eight patients had a change of GLS by more than 15% (three in the dexrazoxane group). However, two patients with reduction of LVEF by more than 5% and GLS change by more than 15% have normal hs-TnT level throughout the entire course of chemotherapy.

**Table 4:** Patients with EF or GLS changes and their peak hs-TnT

Prevention strategy	Sex	Age	Ejection Fraction			Global Strain			Peak hs-TnT
			Pre	C3	End	Pre	C3	End	
Dexrazoxane	M	65	60.5	55	55.2	NA	NA	NA	39.37
Dexrazoxane	F	38	57.1	54.7	50.2	−20.8	−20.4	−16.3	11.01
Dexrazoxane	F	43	70.2	65.3	61.1	−25.9	−21.9	−21.9	10.29
Dexrazoxane	F	49	57.7	51.5	51.6	−21.1	−17.5	−16	4.6
Continuous	M	71	61.3	55.4	55.6	−22.9	−19.1	−15.8	44.07
Continuous	F	24	60.7	53.9	55.3	−20.4	−19.6	−18.1	10.77
Continuous	M	40	57.2	49.9	52	−19.3	−18.1	− 19.3	6.13
Cross Over	F	42	62.3	60.2	55.8	−21	−22.4	−20.1	3.76

*NA* not available, due to technical limitation

## Discussion

Cardiac troponin T is a specific biomarker for myocardial injury. It has been demonstrated that low level elevation of cardiac TnT is associated with histological evidence of myocardial injury induced by doxorubicin [28]. In this study, we measured TnT with highly sensitive assay, which was recently approved by U.S. Food and Drug Administration [29]. The lower limit of detection of the new assay is about 10 times lower than those with the standard assay. In our studies, absolute levels of hs-TnT began to rise at cycle 2 and steadily increase with each cycle of doxorubicin, suggesting the increased hs-TnT is associated with cumulative effect of doxorubicin (Fig. 1). Seven patients who had received cardio-protective strategy had hs-TnT level above detection level of 5 ng/ml, but below 10 ng/ml. Ten patients had a peak hs-TnT level above 10 ng/ml (Table 2). Although the patient numbers are small, it appears that dexrazoxane is less likely to prevent subclinical cardiotoxicity than continuous infusion of doxorubicin. Twelve patients did not have detectable hs-TnT throughout the entire study. It is not known whether these patients can tolerate high dose of doxorubicin. This important clinical issue should be addressed in future studies. Furthermore, echocardiographic parameters appear to be less sensitive in detecting subclinical cardiotoxicity in our study. The long-term consequence of subclinical detection of cardiotoxicity is not known. However, it is clear from this study that better prevention strategy is needed to prevent doxorubicin-induced cardiotoxicity in cancer patients.

### Limitations

This study is conducted only in adult patients with sarcoma. It is not known whether similar results will be obtained from other patient population. However, only sarcoma patients were routinely treated with cardio-protective strategies due to high anticipated doxorubicin exposure. Long-term follow-up will be necessary to determine whether change of EF, GLS or hs-TnT is predictive of late cardiotoxicity. The preventive strategies studied in this paper have not been compared side-by-side with beta-blockers and/or ACE inhibitors. A future study should compare continuous infusion, dexrazoxane, beta-blockers, and ace-inhibitors side-by-side using more sensitive detection methods.

## Conclusion

Using more sensitive detection methods, subclinical doxorubicin-induced cardiotoxicity can still be observed following cardio-protective interventions, such as continuous infusion or dexrazoxane. The consequence of subclinical doxorubicin-induced cardiotoxicity needs to be studied with long-term follow ups. Furthermore, more effective cardio-protection methods should be developed to prevent this dreaded complication of cancer therapy.

## Data Availability

All relevant data are presented in the Methods and Results section.
